# Health trajectories after age 60: the role of individual behaviors and the social context

**DOI:** 10.18632/aging.203407

**Published:** 2021-08-12

**Authors:** Amaia Calderón-Larrañaga, Xiaonan Hu, Miriam Haaksma, Debora Rizzuto, Laura Fratiglioni, Davide L. Vetrano

**Affiliations:** 1 Aging Research Center, Department of Neurobiology, Care Sciences and Society, Karolinska Institutet, Sweden; 2 Department of Public Health and Primary Care, Leiden University Medical Center, The Netherlands; 3 Stockholm Gerontology Research Center, Stockholm, Sweden; 4 Centro di Medicina dell'Invecchiamento, IRCCS Fondazione Policlinico “A. Gemelli”, and Catholic University of Rome, Rome, Italy

**Keywords:** healthy aging, health behaviors, social determinants of health, cohort studies

## Abstract

Background: This study aimed to detect health trajectories after age 60, and to explore to what extent individual and social factors may contribute to healthier aging.

Methods: Twelve-year health trajectories were identified in subjects from the Swedish National Study on Aging and Care in Kungsholmen (N=3108), integrating five indicators of disease, physical and cognitive function, and disability through nominal response models. Growth mixture models were applied to explore health trajectories in terms of rate and pattern of change. Baseline information about health-related behaviors and the social context was collected through standardized questionnaires. The strength of the associations was estimated using logistic regression, and their impact through population attributable fractions (PAF).

Results: Three trajectories were identified grouping 78%, 18%, and 4% of people with respectively increasing rates of health decline. Compared to the best trajectory, subjects in the middle and worst trajectories became functionally dependent 12.0 (95% CI: 11.4-12.6) and 12.1 (95% CI: 11.5-12.7) years earlier, respectively. Insufficient physical activity (OR: 3.38, 95% CI: 2.58-4.42), financial strain (OR: 2.76, 95% CI: 1.77-4.30), <12 years education (OR: 1.53, 95% CI: 1.14-2.04), low social connections (OR: 1.45, 95% CI: 1.09-1.94), low social participation (OR: 1.39, 95% CI: 1.06-1.83) and a body mass index ≥25 (OR: 1.34, 95% CI: 1.03-1.75) were associated with belonging to the middle/worst trajectories. The highest PAFs were observed for insufficient physical activity (27.1%), low education (19.3%) and low social participation (15.9%); a total PAF of 66.1% was obtained.

Conclusions: Addressing the social determinants of health in its broadest sense, complementarily considering life-long factors belonging to the socioeconomic, psychosocial, and behavioral dimensions, should be central to any strategy aimed at fostering health in older age.

## INTRODUCTION

The biological changes underlying the aging process are neither linear nor consistent, and chronological age is an imperfect surrogate measure of this phenomenon. In fact, while some 80-year-olds may enjoy good physical and mental functioning, others may be frail, suffer from a high disease burden, and require significant support in their daily life. Moreover, older people with equal health status at earlier ages may follow different trajectories over time, and their needs will diverge according to which path they are on. Consequently, the health of the older population can be viewed as a continuum and dynamic process that needs to be investigated longitudinally [1].

When looking at trajectories of healthy aging, both what we measure and how we measure it matter equally. Most studies have focused on single impairments or functional limitations [2], however, it is unlikely that any single measure will be capable of reliably capturing the health heterogeneity of aging populations. There is increasing awareness for the need to assess older people’s health in a multidimensional way by using phenotypic indices that capture diseases related to organ systems as well as functional ability, while remaining pragmatically driven by available data in clinical practice and aging studies [3]. Comprehensive assessments of functioning, coupled with measures of more severe disability, are particularly relevant for older adults and are well-aligned with the provision of person-centered care [4]. Moreover, they are much stronger predictors of negative health outcomes than merely the presence of disease [5–7]. The Health Assessment Tool (HAT) used in this study is sensitive to changes in multiple dimensions that people experience as they age. It has a better ability than its individual components in terms of predicting medical and social care needs, self-rated health, or death, and it is based on conventional tests commonly embedded in the Comprehensive Geriatric Assessment (i.e. diseases, physical and cognitive function, and disability) [5, 6, 8].

As for how to measure health changes, capturing distinct developmental health trajectories over time is key, but also a major challenge. Most studies still look at average trajectories of change [9], but more sophisticated techniques, such as latent variable mixture modelling, use multiple long-term observations per individual and allow for improved discernment among alternative health trajectories in terms of rate and pattern of change. In order to apply such techniques, longitudinal data covering different birth cohorts, with low attrition rates, and containing objectively assessed health measures is essential, yet seldom available.

Differences in healthy aging are partly due to genetics and randomly occurring biological dysfunctions, but they are to a great extent influenced by individual health-related behaviors and contextual social determinants (i.e. socioeconomic and psychosocial factors). Despite the strong link of these factors with multimorbidity, frailty, disability, and mortality, [10–14] evidence of their impact on long-term health changes in old age is weaker [15]. Moreover, previous studies have rarely assessed potential risk/protective factors comprehensively, in spite of the increasing recognition of the complex interplay among social and behavioral factors in affecting older individuals’ health status and disease risk [16].

The aims of our study were to identify different health trajectories after age 60, and to explore to what extent health-related behaviors and the social context account for such differences at both the individual and population levels.

## MATERIALS AND METHODS

### Study population

The data were gathered from the longitudinal Swedish National study on Aging and Care in Kungsholmen (SNAC-K) [17]. SNAC-K participants were randomly selected from those who lived at home or in institutions in a central area of Stockholm (Kungsholmen), Sweden, and were older than 60 between 2001-2003, when the study was initiated. The random selection was based on 11 age groups: three younger cohorts (aged 60, 66 and 72) and eight older cohorts (aged 78, 81, 84, 87, 90, 93, 96 and 99+). The younger cohorts were followed every six years and the older ones every three years. In this study, the outcome was assessed using data from the first five follow-up waves (years 2001-2003 to 2013-2015), while the exposures were measured only at baseline.

From an initial study sample of 3,363 individuals (participation rate of 73%), 255 (6.7%) were excluded, as they lacked baseline information on the parameters included in the health score, leaving an analytical sample of 3,108 participants of which 111 were living in nursing homes at baseline (Supplementary Figure 1). Compared to the analytical sample, excluded individuals were significantly older and more likely to be women; with lower education, manual occupation, and financial strain; lower social connections, support, and participation; normal or underweight; non-smokers; and physically inactive (p<0.01).

SNAC-K was approved by the Regional Ethical Review Board in Stockholm, and written informed consent was obtained from participants or their next of kin.

### Multidimensional health assessment

The primary outcome variable was the Health Assessment Tool (HAT) score, which was derived by integrating the following five health indicators objectively assessed during physician and nurse examinations in SNAC-K [5, 6]: 1) Physical function, measured through walking speed; participants were required to walk 6m or 2.44m if they reported walking quite slowly. If the participant was unable to walk, a value of 0 was recorded; 2) Cognitive function, measured by the Mini-Mental State Examination (MMSE); the MMSE score ranges from a minimum value of 0 to a maximum value of 30, with a skewed distribution towards higher scores; 3) Multimorbidity, measured as the number of co-occurring chronic diseases; chronic diseases were defined as those leaving substantial residual disability or impaired quality of life, or requiring a prolonged period of care, treatment, and rehabilitation; [18]; 4) Mild disability, measured as the number out of eight instrumental activities of daily living (IADL) that people were unable to perform independently (grocery shopping, meal preparation, housekeeping, laundry, managing money, using the telephone, taking medications, and using public transportation); and 5) Severe disability, measured as the number out of six basic activities of daily living (ADL) that people were unable to perform independently (bathing, dressing, toileting, continence, transferring, and eating).

A detailed explanation of how HAT is derived can be found in previous articles [5, 6]. Briefly, two parameters, i.e. difficulty and discrimination, were extracted from the regression coefficients of nominal response models after 900 iterations, in order to choose the optimal cut-offs for each health indicator. Because of the high discrimination power of IADL disability, the regression was stratified by limitation status: people with ≤1 impaired IADL, and people with >1 impaired IADL. The HAT score ranges from a minimum value of 0 to a maximum value of 10, with higher values indicating better health. The estimated parameters from baseline data were applied to follow-up waves and used to generate the scores at different time points.

### Exposures related to health behaviors and the social context

Participants’ socioeconomic (i.e. education, occupation, financial strain) and behavioral (i.e. smoking, body mass index (BMI), physical activity) factors were collected during nurses’ interviews. Educational attainment was categorized as elementary, high school, and university or higher; and main occupation as manual or non-manual based on the longest job held during the person's lifetime. Financial strain was measured by asking participants if they had suffered any financial strain within the last year. Smoking was categorized as never, former, and current, and BMI as underweight, normal, overweight, and obese (<18.5 kg/m^2^, 18.5–24.9 kg/m^2^, 25–29.9 kg/m^2^ and ≥30 kg/m^2^, respectively). Physical activity was categorized in three different levels according to intensity [19]: fitness-enhancing (intense exercise several times per week), health-enhancing (light exercise several times per week), and inadequate (≤2-3 times per month of exercise). Psychosocial factors included social connections, support, and participation. Social connections were assessed by asking participants about their marital status, cohabitation status, parenthood, friendships, and the frequency of direct or remote contact with parents, children, relatives, neighbors, and friends [20]. Social support was measured by asking participants about their satisfaction with the aforementioned contacts; perceived material and psychological support; sense of affinity with association members, relatives, and residence area; and being part of a group of friends [20]. Social participation was quantified based on the frequency with which the participants reported attending the theatre, concerts, or art exhibitions; traveling; playing cards/games; or participating in social groups or a pensioners’ organization [20].

### Statistical analysis

HAT score trajectories over 12 years of follow-up were explored using growth mixture models. These models group people into different latent classes, depending on the characteristics of the evolution of HAT scores across time. In other words, growth mixture models enable capturing information about interpersonal differences in intrapersonal change over time. Age was chosen as the time scale to better account for its confounding effect, and a quadratic slope for age was added to account for a potential faster decline of HAT at older ages. The number of latent classes was chosen based on the Bayesian Information Criterion (BIC) and the Lo-Mendell-Rubin (LMR) likelihood ratio test. The inclusion of random effects and whether these were class-specific or class-invariant was decided based on the likelihood ratio test (LRT). The variance of the quadratic slope was fixed to zero and the residual variances, which were assumed to be equal across classes, were allowed to vary across age groups. Growth mixture models were run for the general population as well as for age and sex subgroups. Subjects were assigned to the latent class to which they had the highest probability of belonging. Given the small size of one of the three obtained classes, two of them (i.e. the middle and worst trajectories) were combined.

Logistic regression models were applied to identify possible determinants of HAT trajectories (i.e. classes). The adjustment of the models was conducted in three steps: 1) baseline age, sex, and each determinant, individually (model I); 2) baseline age, sex, and all variables belonging to each domain, separately (i.e. socioeconomic, psychosocial and behavioral factors) (model II); 3) baseline age, sex, and all determinants, simultaneously (model III). Interactions between age, sex, and each of the socioeconomic, psychosocial and behavioral variables were also analyzed. Pairwise interactions among all socioeconomic, psychosocial, and behavioral variables were also tested in model III. The most favorable class (i.e. for trajectories) and category (i.e. for the determinants) were chosen as the reference. In addition, Laplace regression was applied to estimate differences in time to functional dependency (i.e. HAT score <5) and to death among different HAT trajectories.

Last, population attributable fractions (PAFs) were calculated for those determinants showing a statistically significant association in the logistic regressions. The PAF represents the proportion of the adverse outcome that would be avoided if an individual risk factor could be fully eliminated and, given its interpretation, it is well suited for public policy making [21]. The calculation of the PAF is based on both the magnitude of the association of a given outcome with one or more exposures, as well as the prevalence of the exposure(s) in the population. Because the interpretation of PAFs is more straightforward with binary exposures, all exposure variables were dichotomized as follows: elementary/high school vs. university education; manual vs. non-manual occupation; financial strain yes vs. no; low vs. middle/high social connections; low vs. middle/high social support; low vs. middle/high social participation; current/former vs. never smoker; obese/overweight vs. normal/underweight BMI; and inadequate vs. fitness-/health-enhancing physical activity. Group-level PAFs were also calculated for those determinants belonging to the same domain (i.e. socioeconomic, psychosocial and behavioral factors), where only those variables that showed a significant association in the domain-level logistic regressions (model II) were included. Last, overall PAFs were calculated for those determinants that showed a significant association in the final multivariate model (model III).

Growth mixture models were fitted using MPlus version 8.4; nominal response models, logistic and Laplace regressions were performed using Stata version 15.1; and results were processed using R version 3.6.1.

### Data sharing

Data are from the SNAC-K project, a population-based study on aging and dementia (http://www.snac-k.se/). Access to these original data is available to the research community upon approval by the SNAC-K data management and maintenance committee. Applications for accessing these data can be submitted to Maria Wahlberg (Maria.Wahlberg@ki.se) at the Aging Research Center, Karolinska Institutet.

## RESULTS

**Figure 1:**
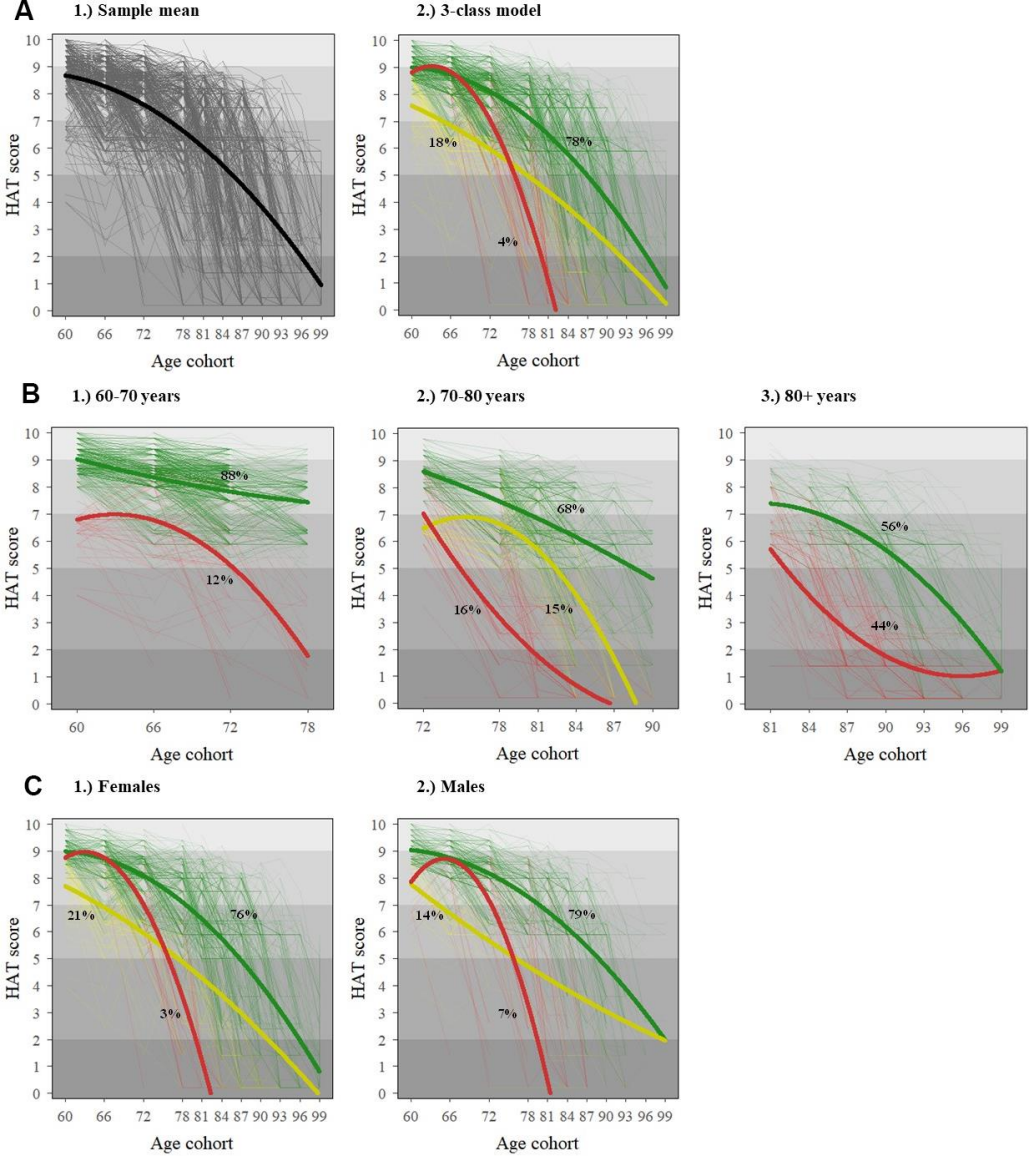
Individual (dotted) and average (solid) HAT-score trajectories for the general population (**A**) and stratified by baseline age (**B**) and sex (**C**). The x-axis in all graphs reflects the age structure of SNAC-K, whereby participants belong to any of the following 11 age cohorts (aged 60, 66, 72, 78, 81, 84, 87, 90, 93, 96 and 99+) both at baseline and at follow-ups. The shading in the background of the graphs reflects the clinical interpretation of HAT scores, as described in Table 1.

The distribution of all demographic, socioeconomic, psychosocial, and behavioral variables for the total study sample is shown in Supplementary Table 1. In Table 1, the most prevalent values for each health indicator by baseline HAT score are shown. Figure 1A shows the results from the best-fit growth mixture model and the sample mean curve for the general population; 78% of the population was included in the best trajectory (i.e. green), with a faster decline from age 80 onwards; 18% of the population belonged to the middle trajectory (i.e. yellow) with lower initial HAT scores and a similar decline between 60-80 years, but a slower decline from age 80 onwards; and 4% of the population was categorized in the worst trajectory (i.e. red) with similar initial scores as the best trajectory but a much faster decline. For example, on average, a person aged 60 years in the green trajectory would decline 2.3 points in the HAT score across the subsequent 20 years, while a person of the same age in the red trajectory would decline 6.9 points across the same time period, even if both persons had similar HAT scores at age 60. Results from Laplace regressions showed that, compared to the best trajectory, subjects in the middle and worst ones lost their functional independence (i.e. HAT score <5) 12.0 (95% CI: 11.4-12.6) and 12.1 (95% CI: 11.5-12.7) years earlier, respectively. The age- and sex-adjusted median survival of subjects in the middle and worst trajectories was 4.28 (95% CI: 3.51-5.06) and 3.51 (95% CI: 2.52-4.50) years shorter than those belonging to the best trajectory, respectively.

**Table 1:** Most frequent values (i.e. prevalence above 50%) for each health indicator, by HAT score.

**HAT score**	**ADL limitations**	**IADL limitations**	**Walking speed**	**Number of CD**	**MMSE**
0.0-0.9	0	0-1	≥1.5	1	30
1.0-1.9	0	0-1	≥1.5	2-4	30
2.0-2.9	0	0-1	1.5-1.0	2-4	30-29^*^
3.0-3.9	0	0-1	1.5-1.0	5+	28-20
4.0-4.9	0	0-1	1.0-1.4	2-4	30-29
5.0-5.9	0	0-1	1.0-0.4	5+	28-20
6.0-6.9	0	2+	1.0-0.4	5+	30-29^*^
7.0-7.9	0	2+	1.0-0.4	5+	28-20
8.0-8.9	0	2+	<0.4	5+	28-20
9.0-9.4	1+	2+	<0.4	5+	28-20
9.5-10	1+	2+	<0.4	5+	19-0

HAT, Health Assessment Tool; ADL, basic activities of daily living; IADL, instrumental activities of daily living; CD, chronic diseases; MMSE, Mini-Mental State Examination.

Clinical interpretation of HAT scores: 0-1.9 = severe functional dependence; 2-4.9 = mild functional dependence; 5-6.9 = compromised physical functioning with multimorbidity and medium cognitive functioning; 7-8.9 = light decreased physical or cognitive functioning with some morbidities; 9-10 = good physical functioning and morbidity status.

^*^As no category was above 50%, the two with highest prevalence are reported.

Figure 1B, 1C show the results from the best-fit growth mixture models stratified by baseline age and sex, respectively. While only two trajectories were identified in the groups aged 60-70 (n=1251) and 80+ years (n=975) at baseline, three trajectories were identified in the group aged 70-80 years (n=882) at baseline. Three trajectories were also found for both males and females in the sex-stratified models. The best (i.e. green) and middle (i.e. yellow) trajectories showed steeper declines in females compared to males. Yet, the prevalence of the worst trajectory (i.e. red) was double in males compared to females. The parameter estimates and fitting criteria as well as their interpretation for all these models can be found in Supplementary Tables 2, 3.

Table 2 shows the odds ratios and 95% CI for all potential socioeconomic, psychosocial, and behavioral determinants. For model I (adjusted by baseline age, sex, and each variable individually), all factors were significantly associated with belonging to the middle/worst vs. best trajectory, but occupation, smoking, and social support lost their statistical significance in model II, which was further adjusted by other domain-specific factors. The effect of physical activity was largely attenuated in model III, when all factors were simultaneously adjusted for. Among all possible pairwise interactions, only those between manual occupation and high BMI (OR: 2.10, 95% CI: 1.06-4.15) and between manual occupation and low social connections (OR: 0.46, 95% CI: 0.23-0.90) were significant. The correlation matrix among all exposure variables is shown in Supplementary Table 4, and the odds ratios and 95% CI for the non-dichotomized exposures in Supplementary Table 5.

**Table 2:** Odds ratios (OR) and 95% confidence intervals (95% CI) for belonging to the middle/worst vs. best trajectory.

	**Model I**	**Model II**	**Model III**
	**OR (95% CI)**	**OR (95% CI)**	**OR (95% CI)**
**Socioeconomic factors**			
Education (elementary/high school vs. university)	1.87 (1.47-2.37)	1.84 (1.43-2.38)	1.53 (1.14-2.04)
Occupation (manual vs. non-manual)	1.54 (1.20-1.97)	1.26 (0.97-1.64)	1.13 (0.81-1.57)
Financial strain (yes vs. no)	2.80 (1.95-4.02)	2.67 (1.85-3.84)	2.76 (1.77-4.30)
**Psychosocial factors**			
Social connections (low vs. middle/high)	1.88 (1.47-2.39)	1.59 (1.21-2.09)	1.45 (1.09-1.94)
Social support (low vs. middle/high)	1.67 (1.32-2.12)	1.29 (0.98-1.69)	1.05 (0.78-1.40)
Social participation (low vs. middle/high)	1.56 (1.21-2.02)	1.52 (1.17-1.97)	1.39 (1.06-1.83)
**Behavioral factors**			
Smoking (current/former vs. never)	1.29 (1.04-1.60)	1.20 (0.95-1.52)	1.20 (0.92-1.57)
BMI (obese/overweight vs. normal/underweight)	1.37 (1.10-1.72)	1.28 (1.01-1.61)	1.34 (1.03-1.75)
Physical activity (inadequate vs. fitness-/health-enhancing)	4.90 (3.90-6.16)	4.28 (3.38-5.44)	3.38 (2.58-4.42)

Model I adjusted by baseline age, and sex.

Model II adjusted by baseline age, sex and all domain-specific factors.

Model III adjusted by baseline age, sex and all other factors.

In stratified analyses, while the risk effect of low education, occupation, and financial strain; high BMI; and low social connections seemed to decrease with age, it increased for smoking and inadequate physical activity. Financial strain (OR: 4.33, 95% CI: 1.45-12.99) and inadequate physical activity (OR: 3.57, 95% CI: 2.59-4.91) were the factors with the highest risk effect for the youngest and oldest groups, respectively (Supplementary Figure 2A). Furthermore, the effect of financial strain was higher for males (OR: 3.18, 95% CI: 1.54-6.59) than for females (OR: 2.55, 95% CI: 1.46-4.44). However, the opposite was true for physical activity (OR: 3.56, 95% CI: 2.53-5.00 for females; OR: 3.11, 95% CI: 2.02-4.78 for males). Social participation was only significant for females (OR: 1.55, 95% CI: 1.10-2.20, Supplementary Figure 2B).

The study of the PAFs, as shown in Table 3, revealed that insufficient physical activity was the most important contributor (PAF: 27.1%, 95% CI: 20.4%-33.3%) for belonging to the middle/worst vs. best trajectory, followed by low education (PAF: 19.3%, 95% CI: 5.7%-31.0%) and low social participation (PAF: 15.9%, 95% CI: 2.2%-27.7%). Behavioral factors showed the highest domain-specific PAFs (PAF: 37.4%, 95% CI: 26.1%-47.1%). Socioeconomic (PAF: 24.7%, 95% CI: 11.3%-36.0%) and psychosocial factors (PAF: 23.8%, 95% CI: 9.3%-35.9%) had similar domain-specific PAFs, with percentages around two-thirds of that for behavioral factors. In total, 66.1% (95% CI: 54.1%-75.0%) of people in the middle/worst trajectories could potentially belong to the best trajectory, if all significant risk factors were eliminated simultaneously.

**Table 3:** Factor-level, domain-level, and overall population attributable fractions (PAFs; middle/worst vs. best trajectory).

	**Individual PAFs (95% CI)**	**Domain PAFs (95% CI)**
**Socioeconomic factors**		
Education (elementary/high school vs. university)	19.3% (5.7%-31.0%)	24.7% (11.3%-36.0%)
Occupation (manual vs. non-manual)	---	
Financial strain (yes vs. no)	6.1% (2.9%-9.1%)	
**Psychosocial factors**		
Social connections (low vs. middle/high)	9.0% (1.7%-15.8%)	23.8% (9.3%-35.9%)
Social support (low vs. middle/high)	---	
Social participation (low vs. middle/high)	15.9% (2.2%-27.7%)	
**Behavioral factors**		
Smoking (current/former vs. never)	---	37.4% (26.1%-47.1%)
BMI (obese/overweight vs. normal/underweight)	13.2% (0.7%-24.1%)	
Physical activity (inadequate vs. fitness-/health-enhancing)	27.1% (20.4%-33.3%)	
	**Overall PAF (95% CI)**	66.1% (54.1%-75.0%)

PAFs were obtained based on logistic regression adjusted for age, sex and all other factors (model III), and only for those variables showing a significant association.

## DISCUSSION

In this study aiming to explore health changes in an older urban Swedish population, we identified three trajectories with marked health differences both at age 60 and over time, with 78%, 18%, and 4% of the study participants belonging to the best, middle, and worst trajectories, respectively. A mix of health-related behavioral factors (e.g. physical activity) and contextual social factors (e.g. education and social participation) contributed mostly to the levels and rates of change of health trajectories. In an extreme scenario where all associated life-long factors were eliminated, 66% of those in the middle and worst trajectories could potentially follow the same slow health decline as older adults belonging to the best trajectory.

Previous studies comparing trajectories of health changes over time have investigated the development of single-domain disorders, such as chronic diseases [22, 23] or disabilities [24, 25]. In this study we were able to incorporate several of these measures into a composite metric of healthy aging, truly reflecting people′s capacity to do what they value most, which is being able to move around, take care of themselves, and decide independently [1]. When looking at changes over time, we found that, compared to the best trajectory, which comprised approximately three quarters of the study population, subjects in the middle and worst trajectories became functionally dependent 12 years earlier, around the age of 78 years. A similar time gap was found between centenarians in SNAC-K, an example of successful agers, and their shorter-lived cohort counterparts concerning the occurrence of multimorbidity, disability, and cognitive impairment [26].

The age- and sex-stratified health trajectories found in our study were comparable to those seen in the general population, and further unraveled some interesting traits. In terms of age, we found that the health heterogeneity widens considerably in the 70 to 80 years' decade, as indicated by the increase in the number of classes identified in this age range. While previous research from our group [27] and others [28] has shown that until age 80, most people are free from functional impairment or disability despite the presence of chronic disorders, age 70 seems to be a key period when minority groups start to significantly deviate from average trends. The lower number of health trajectories identified in octogenarians and nonagenarians may be due to survival selection characterizing individuals reaching old ages. In terms of sex, females in the best and middle trajectories exhibited steeper health declines than males, which is in line with the disability-survival paradox whereby women suffer more frequently from nonfatal but disabling diseases, whereas men have fewer diseases in total but more of these are life-threatening [29].

The impact of socioeconomic, psychosocial, and behavioral factors on health changes in old age has already been examined, but most past work focused on single exposures and specific health dimensions [23, 25]. According to a recent systematic review on modifiable risk factors for healthy aging (understood as a multidimensional construct) by Kralj et al. [15], educational level is the most frequently explored socioeconomic determinant, with the majority of studies reporting a beneficial association between having higher levels of education and healthier aging. Consistently, elementary/high school vs. university education accounted for 19% of all cases belonging to the middle and worst trajectories; the highest PAF value among all studied determinants. However, we found an even stronger association between financial strain and belonging to the middle/worst vs. best trajectory. Besides entailing a better economic status, higher education may enable a more effective use of power, money, general and health-related literacy, prestige, or social support to protect oneself from health risks or mitigate the consequences of poor health [30]. Our results suggest that older people’s health is partly shaped by material factors such as income and wealth, which allow people to secure goods and services needed for a healthy life (e.g. housing, food, healthcare), but also by non-material resources and capabilities afforded by education. Our findings differ from two previous longitudinal multi-cohort aging studies, where education and wealth were consistently associated with cross-sectional health differences in old age, but not with their evolution over time [31, 32]. Notably, both these studies focused on average health changes over time, without taking interindividual differences into consideration.

Findings on the association between occupation and healthy aging remain inconclusive [15]. In our study, the association was no longer significant after including education and financial strain into the models. This is probably because, unlike the measures of wealth that reflect a process of life-long accumulation, occupation refers to more distant periods of time already accounted for by education.

More than one third of cases belonging to the middle and worst trajectories could be attributed to health-related behavioral determinants including BMI and physical activity (i.e. PAF: 37%). Despite being clearly influenced by economic and cultural status, health behaviors are often analyzed separately from other social determinants [16]. Inadequate levels of physical activity were strongly associated with belonging to poorer health trajectories in our study, and its effect size increased with increasing age. This is in line with studies indicating that, even in very advanced years, physical activity can have powerful benefits for health improvement and well-being [33, 34]. It is also in agreement with a recent study that found that health behaviors, even more than socioeconomic status, play a major role in the risk of dying among subjects with cardiometabolic multimorbidity [35]. Despite such evidence on the health benefits of physical activity, we are aware that reverse causation may also play a role in explaining our strong association, even if the longitudinal design of our study should help minimize this possibility. Moreover, previous population-based longitudinal research has shown reasonable stability in physical activity performance among older adults [36].

As in the present study, a consistent negative association between higher BMI in late life and healthy aging has been reported across the literature [15]. Data from the Whitehall II study suggests that the odds of aging successfully with a BMI ≥30 is almost three times lower compared to someone of normal weight [37]. The decreasing effect size of BMI at older ages could be related to its different clinical meaning in the youngest versus oldest old. In fact, a U-shaped association between BMI and mobility decline, disability, and all-cause mortality has been previously described among the oldest old [38]. In old age, a lower BMI may reflect sarcopenia, defined as the reduction in muscle mass that increases subjects’ risk of falls, disability, and mortality [39, 40]. Moreover, low BMI may indicate malnutrition and can be an expression of chronic disease severity [41].

As for smoking, the significance of the association between current smoking and belonging to a poorer health trajectory was lost when further adjusting for the rest of the behavioral factors, even though the direction was consistent throughout all models. According to the systematic review by Kralj et al. [15], up to 25% of studies reported a non-significant negative association of smoking with health changes in older age. This may be due to the health selection seen for current smokers across aging cohorts, even if, in our study, no significant differences were seen between age groups concerning the strength of the association.

Psychosocial determinants such as social connections, support, and participation are increasingly relevant to older people's views of successful aging [42]. It has been shown that these affect health via pathways such as provision of help with daily activities, material assistance, advice and information on particular needs, and cognitive and emotional stimulation [43]. Social relationships may also buffer the negative impact of stress on health [44]. In our study, a lower participation in social activities was the psychosocial factor most strongly associated with worse health trajectories, which is supported by a recent study looking at older people’s participation in community groups [45]. Women received a stronger benefit from social participation than men, which may indicate that more and different aspects related to social capital impact healthy aging among women than men [46].

Social connections, the second strongest psychosocial determinant, have consistently shown a strong protective association with health in old age [15]. Our results also revealed a significant association between low social connections and worse health trajectories, but the association became weaker in older age groups. This is probably due to the socio-selectivity theory whereby, as the end of life approaches, priorities shift from network size and diversity towards emotional components [47]. As for social support, the association with health trajectories lost significance in the fully adjusted model even if its direction was consistent across models, most likely due to the high correlation between social support and connections.

Considering that aging populations will comprise a growing proportion of our global world, we need to adapt the ways we live to maximize the opportunities that longevity presents. The COVID-19 pandemic has unraveled how vulnerable aging populations can become when faced with unexpected threats, highlighting the need to shift in the way we think about global aging. Ours and other studies provide evidence that can guide societies to develop healthy, empowered and resilient communities of older people throughout the life course. Of the behavioral and contextual factors assessed, the largest numbers of cases within the worse trajectories were potentially attributable to physical activity, education and social participation, suggesting that the most effective public health strategies and campaigns for ensuring healthy aging in Sweden should focus on enhancing the opportunities to build up on these factors throughout life. Still, the prioritization of the different socioeconomic, psychosocial, and behavioral determinants on which to act upon may vary by country or community, depending on their prevalence and strength of association with the health trajectories. Future research should inform when these interventions should take place throughout the life span, in order to optimize their effectiveness.

### Study limitations

The primary limitation of this study is that our findings are likely to apply only to wealthy settings with universal healthcare. Indeed, SNAC-K participants are fitter and wealthier than the average Swedish older population. SNAC-K may be considered as the best-case scenario beyond which health trajectories as well as their association with potential risk factors would likely emerge as steeper and stronger, respectively. What is interesting is that, even within an exceptionally well-off population as SNAC-K, considerable heterogeneity remains in terms of how older people’s health evolves over time, and well-known behavioral and contextual factors seem to explain such variation to an important extent. All determinants were measured at baseline, when SNAC-K participants belonged to different age groups, which assumes that all these factors are relatively stable over time. However, by measuring the exposures only at baseline, we were better able to untangle the chronology of exposures and the outcome, thus decreasing the risk of reverse causality. Several assumptions underlie the PAF, the most important being that the estimated effect is adjusted for all confounders. In this sense, relevant behavioral factors such as diet, alcohol intake, sedentary behaviors or sleep patterns are purposefully missing from this study given the difficulty to obtain reliable measures. Moreover, PAFs also assume that removing the exposure does not affect other risk factors, which may not always be true in practice. While attrition due to death or dropout can introduce bias in aging cohort studies, these data were considered to provide useful prognostic information in relation to the health trajectories, and were thus analyzed as a separate study outcome. Last, in order to truly capture the experience of healthy aging, future studies will necessarily need to focus on multiple time points throughout the life course, beyond those examined in this study [48].

## CONCLUSIONS

Poor health is not an inevitable consequence of survival to older ages. A number of factors linked to individuals’ health-related behaviors and social context, such as physical activity, financial strain, social participation, education, social connections, and BMI lead to important variations in older peoples’ health trajectories. By intervening on all these factors throughout life, up to 66% of people in the poorest health trajectories could be steered towards a healthier aging. Addressing the social determinants of health in its broadest sense, complementarily considering life-long factors belonging to the socioeconomic, psychosocial, and behavioral dimensions, should be central to any strategy aimed at fostering health in older age.

## Supplementary Material

Supplementary Figures

Supplementary Tables
