# Supplementary material for: Health trajectories after age 60: the role of individual behaviors and the social context
Source: Aging (Albany NY). 2021 Aug 12;13(15):19186–206. doi: 10.18632/aging.203407 (PMC8386565; doi:10.18632/aging.203407)
Supplement: Supplementary Figures [file aging-13-203407-s001.pdf]

SUPPLEMENTARY FIGURES

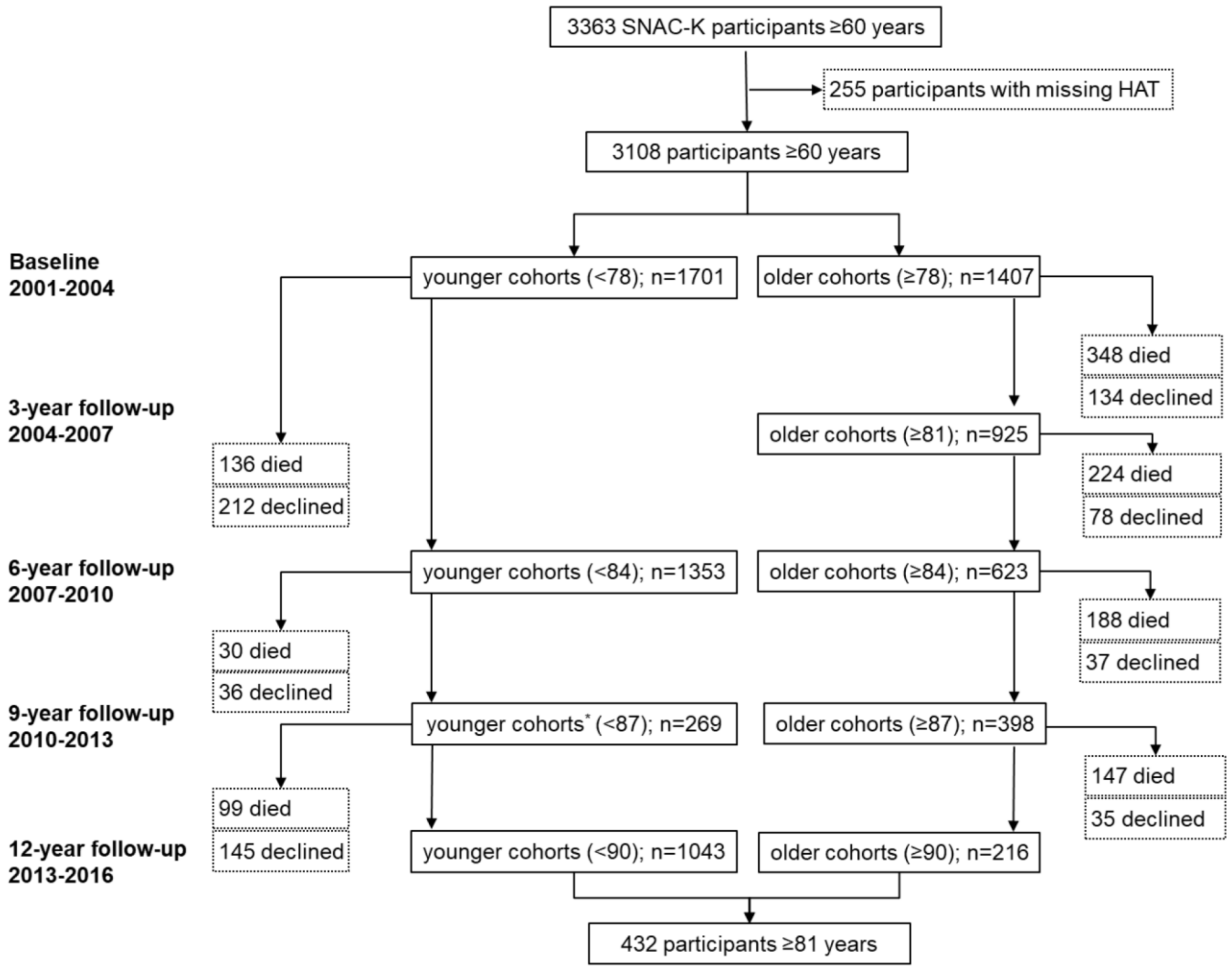

Supplementary Figure 1. Flow-chart of the study population. *Only includes participants who belonged to the age cohort that was 72 years old at baseline.

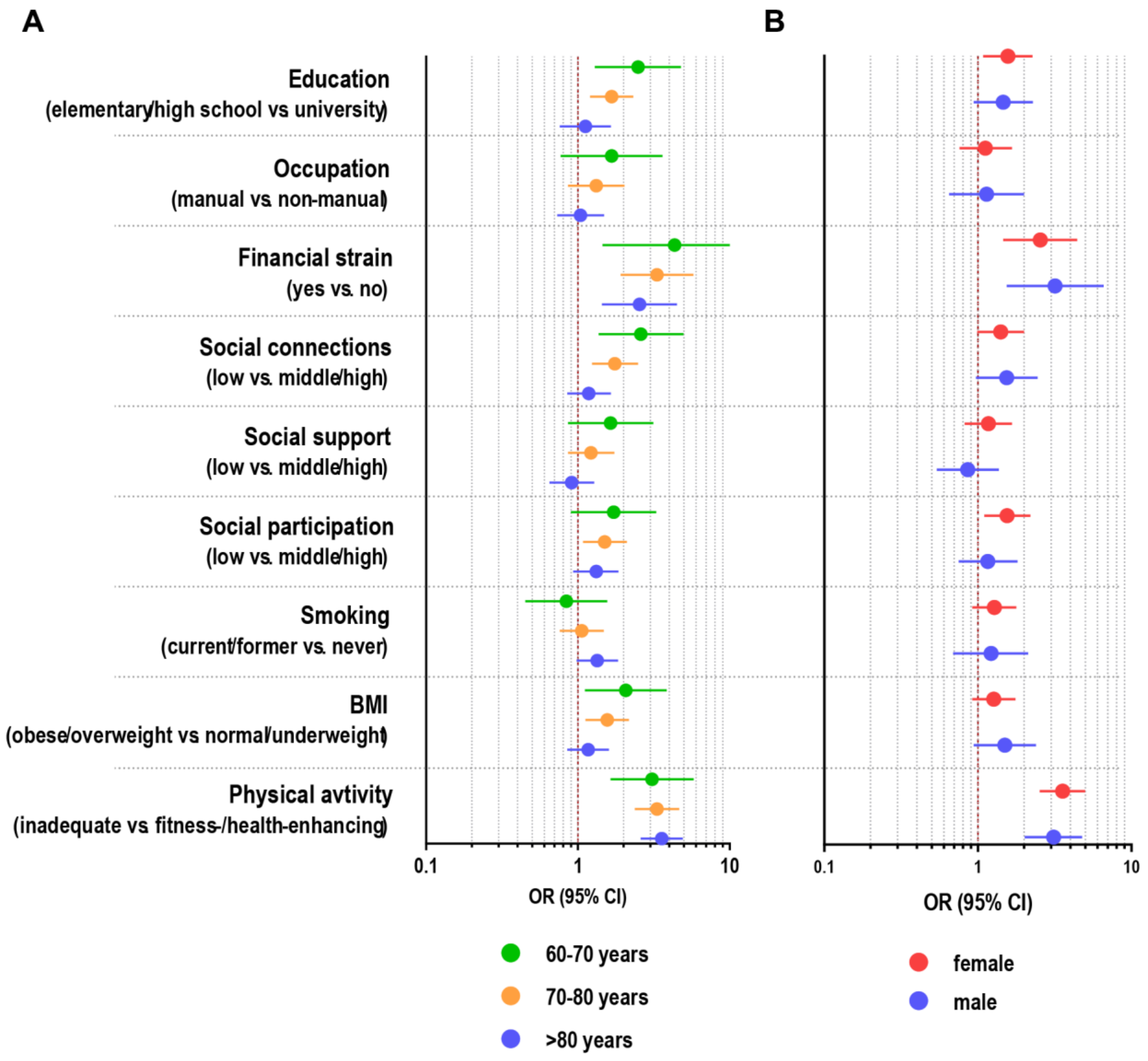

Supplementary Figure 2. Odds ratios (OR) and 95% confidence intervals (95% CI) for belonging to the middle/worst vs. best trajectory, stratified by baseline age (**A**) and sex (**B**). Models are mutually adjusted for all exposures. OR: odds ratio; CI: confidence interval. p-values for interactions of age with education=0.067; occupation=0.275; financial strain=0.441; smoking=0.209; BMI=0.120; physical activity=0.696; social connections=0.039; social support=0.131; social participation=0.511. p-values for interactions of sex with education=0.809; occupation=0.957; financial strain=0.632; smoking=0.496; BMI=0.566; physical activity=0.630; social connections=0.757; social support=0.282; social participation=0.312.
