# Supplementary material for: Health trajectories after age 60: the role of individual behaviors and the social context
Source: Aging (Albany NY). 2021 Aug 12;13(15):19186–206. doi: 10.18632/aging.203407 (PMC8386565; doi:10.18632/aging.203407)
Supplement: Supplementary Tables [file aging-13-203407-s002.pdf]

SUPPLEMENTARY TABLES

Supplementary Table 1. Descriptive statistics for the total population and by the three HAT-score trajectories obtained in the growth mixture model.

	Total	Green	Yellow	Red
	n=3108	n=2667 (85.8%)	n=374 (12.0%)	n=67 (2.2%)
**Average age at baseline, mean (SD)**	74.18 (10.96)	74.75 (11.14)	70.62 (9.56)	71.34 (5.41)
**Females, n (%)**	1995 (64.19)	1704 (63.89)	254 (67.91)	37 (55.22)
**Socioeconomic factors**				
**Education, n (%)**				
Elementary	526 (16.92)	441 (16.54)	73 (19.52)	12 (17.91)
High school	1529 (49.20)	1298 (48.67)	196 (52.41)	35 (52.24)
University	1041 (33.49)	920 (34.5)	101 (27.01)	20 (29.85)
**Occupation, n (%)**				
Manual worker	724 (23.29)	612 (22.95)	96 (25.67)	16 (23.88)
Non-manual worker	2347 (75.51)	2035 (76.30)	261 (69.79)	51 (76.12)
**Financial strain, n (%)**				
Yes	163 (5.24)	115 (4.31)	45 (12.03)	3 (4.48)
No	2859 (91.99)	2500 (93.74)	296 (79.14)	63 (94.03)
**Psychosocial factors**				
**Social connections, n (%)**				
Low	946 (30.44)	804 (30.15)	119 (31.82)	23 (34.33)
Medium	946 (30.44)	828 (31.05)	97 (25.94)	21 (31.34)
High	946 (30.44)	851 (31.91)	76 (20.32)	19 (28.36)
**Social support, n (%)**				
Low	948 (30.50)	810 (30.37)	124 (33.16)	14 (20.90)
Medium	968 (31.15)	847 (31.76)	94 (25.13)	27 (40.30)
High	925 (29.76)	828 (31.05)	75 (20.05)	22 (32.84)
**Social participation, n (%)**				
Low	1623 (52.22)	1405 (52.68)	183 (48.93)	35 (52.24)
Medium	626 (20.14)	561 (21.03)	50 (13.37)	15 (22.39)
High	356 (11.45)	322 (12.07)	25 (6.68)	9 (13.43)
**Behavioral factors**				
**Smoking, n (%)**				
Current	435 (14.00)	341 (12.79)	82 (21.93)	12 (17.91)
Former	1179 (37.93)	1018 (38.17)	131 (35.03)	30 (44.78)
Never	1443 (46.43)	1279 (47.96)	140 (37.43)	24 (35.82)
**BMI, n (%)**				
Obese	368 (11.84)	274 (10.27)	83 (22.19)	11 (16.42)
Overweight	1155 (37.16)	1012 (37.95)	114 (30.48)	29 (43.28)
Underweight	81 (2.61)	68 (2.55)	12 (3.21)	1 (1.49)
Normal weight	1288 (41.44)	1151 (43.16)	113 (30.21)	24 (35.82)
**Physical activity, n (%)**				
Inadequate	1001 (32.21)	760 (28.50)	223 (59.63)	18 (26.87)
Health-enhancing	1471 (47.33)	1317 (49.38)	117 (31.28)	37 (55.22)
Fitness-enhancing	636 (20.46)	590 (22.12)	34 (9.09)	12 (17.91)

SD: standard deviation; BMI: body mass index.

All proportions are significantly different among the three trajectories.

Divergences between total numbers and the final sample size (n=3108) is due to missing data. Missing data for education: 12; occupation: 37; financial strain: 86; smoking: 51; BMI: 216; social connections: 270; social support: 267; social participation: 503.

The colors indicated in each column correspond to those used to depict the trajectories in Figure 1A–1C.

Supplementary Table 2. Parameter estimates for HAT-score trajectories by latent class for the general population and by groups of age and sex.

	Green	Yellow	Red
**General population**			
Prevalence, n (%)#	2667 (85.8%)	374(12.0%)	67 (2.2.%)
Fixed effects, mean (SE)			
Intercept (age=60)	9.00 (0.03)**	7.58 (0.18)**	8.80 (0.26)**
Linear rate of change	-0.01 (0.01)*	-0.11 (0.03)**	0.16 (0.05)**
Quadratic rate of change	-0.01 (0.00)**	-0.002 (0.001)*	-0.03 (0.00)**
Random effects, mean (SE)			
Intercept variance	0.02 (0.05)	1.66 (0.26)**	0.00 (0.13)
Slope variance	0.002 (0.000)**	0.006 (0.001)**	0.03 (0.01)*
**60-70 years**			
Prevalence, n (%)#	1135 (90.7%)	---	116 (9.3%)
Fixed effects, mean (SE)			
Intercept (age=60)	9.03 (0.02)**	---	6.80 (0.18)**
Linear rate of change	-0.12 (0.008)**	---	0.14 (0.07)*
Quadratic rate of change	0.002 (0.001)**	---	-0.02 (0.005)**
Random effects, mean (SE)			
Intercept variance	0.15 (0.05)**	---	2.01 (0.40)**
Slope variance	0.001 (0.001)	---	0.03 (0.01)**
**70-80 years**			
Prevalence, n (%)#	657 (74.5%)	107 (12.1%)	118 (13.4%)
Fixed effects, mean (SE)			
Intercept (age=70)	8.92 (0.05)**	5.79 (0.16)**	8.63 (0.27)**
Linear rate of change	-0.16 (0.02)**	0.42 (0.08)**	-0.84 (0.07)**
Quadratic rate of change	-0.003 (0.001)*	-0.04 (0.006)**	0.02 (0.004)**
Random effects, mean (SE)			
Intercept variance	0.33 (0.14)*	0.14 (0.15)	4.25 (0.76)
Slope variance	0.01 (0.002)**	0.009 (0.002)**	0.009 (0.002)**
**80+ years**			
Prevalence, n (%)#	549 (56.3%)	---	426 (43.7%)
Fixed effects, mean (SE)			
Intercept (age=80)	7.41 (0.15)**	---	6.36 (0.27)**
Linear rate of change	-0.003 (0.04)	---	-0.67 (0.04)**
Quadratic rate of change	-0.02 (0.002)**	---	0.02 (0.002)**
Random effects, mean (SE)			
Intercept variance	0.97 (0.26)**	---	2.87 (0.36)**
Slope variance	0.025 (0.004)**	---	0.005 (0.003)*
**Females**			
Prevalence, n (%)#	1697 (85.1%)	271 (13.6%)	27 (1.4%)
Fixed effects, mean (SE)			
Intercept (age=60)	8.99 (0.04)**	7.70 (0.21)**	8.75 (0.53)**
Linear rate of change	-0.02 (0.01)*	-0.12 (0.04)**	0.14 (0.11)
Quadratic rate of change	-0.005 (0.000)**	-0.002 (0.001)*	-0.02 (0.01)**
Random effects, mean (SE)			
Intercept variance	0.03 (0.07)	1.62 (0.35)**	0.19 (0.35)
Slope variance	0.003 (0.001)**	0.005 (0.002)**	0.02 (0.01)
**Males**			
Prevalence, n (%)#	948 (85.2%)	117 (10.5%)	48 (4.3%)
Fixed effects, mean (SE)			

Intercept (age=60)	9.04 (0.04)**	7.78 (0.37)**	7.84 (0.51)**
Linear rate of change	-0.03 (0.01)*	-0.19 (0.05)**	0.34 (0.09)**
Quadratic rate of change	-0.004 (0.000)**	0.001 (0.002)	-0.03 (0.004)**
Random effects, mean (SE)			
Intercept variance	0.10 (0.07)	2.21 (0.66)**	1.28 (0.74)*
Slope variance	0.002 (0.001)**	0.006 (0.002)**	0.07 (0.03)*

#Reported class counts and proportions are based on individuals' most likely class membership.

Significance level for the fixed and random effects: *p<0.05; **p<0.01.

Interpretation of parameter estimates taking the general population as an example: the mean coefficients for the green trajectory are 9.00, -0.01, and -0.01 for the intercept at age 60, linear rate of change, and quadratic rate of change, respectively, which means that the trajectory starts at a HAT score of 9.00 at age 60, with an annual linear decline of 0.01 and a quadratic annual decline of 0.01 in the HAT score with increasing age (e.g. at age 62, the quadratic decline will be of 0.01*2*2=0.04). The quadratic coefficient for the red trajectory is three times higher (and still statistically significant) compared to the green trajectory (i.e. -0.03), which reflects a much faster decline for the red trajectory, as shown in Figure 1.A.2. Variances of the random effects represent the extent to which individuals are deviant from the mean of the respective trajectory, which is represented through the individual observed HAT-score trajectories (i.e. dotted lines) in Figure 1.

The colors indicated in each column correspond to those used to depict the trajectories in Figure 1A–1C.

Supplementary Table 3. Overview of model fit criteria during class enumeration for the general population and by groups of age and sex.

# classes	# parameters	BIC	Entropy	LMR p-value	Smallest class size (%)*
**General population**					
Step1					
1	14	28860.591	---	---	---
2	18	27331.979	0.803	0.000	16.6
3	22	26856.519	0.675	0.090	5.8
4	26	26569.106	0.630	0.000	1.1
Step2					
1	17	27234.634	---	---	
2	23	26454.085	0.545	0.000	14.6
3[a]	29	26369.050	0.665	0.000	2.2
**60-70 years**					
Step1					
1	7	10059.603	---	---	---
2	11	9313.044	0.944	0.000	8.5
3	15	9169.783	0.880	0.284	6.5
4	19	8900.17	0.908	0.001	1.4
Step2					
1	10	9618.147	---	---	
2	16	8899.77	0.833	0.000	9.3
3	22	8750.12	0.824	0.063	7.8
**70-80 years**					
Step1					
1	9	10387.4	---	---	---
2	13	9680.678	0.81	0.000	19.0

3	17	9446.913	0.638	0.001	14.5
4	21	9322.693	0.708	0.001	1.9
Step2					
2	17	9262.994	0.775	---	14.5
3[a]	22	9147.952	0.746	0.000	12.1
**80+ years**					
Step1					
1	10	8424.815	---	---	---
2	14	7890.327	0.653	0.000	41.3
3	18	7764.03	0.558	0.029	22.9
4	22	7653.369	0.572	0.026	10.9
Step2					
1	13	7857.903	---	---	
2	19	7633.289	0.631	0.000	43.7
3	25	7649.23	0.478	0.620	13.5
**Females**					
Step1					
1	14	18982.422	---	---	---
2	18	18060.241	0.748	0.011	21.1
3	22	17740.017	0.555	0.001	13.1
4	26	17589.537	0.622	0.000	1.0
Step2					
2	23	17525.13	0.499	---	16.2
3[b]	29	17520.03	0.655	0.064	1.4
**Males**					
Step1					
1	14	9909.252	---	---	---
2	18	9322.168	0.877	0.007	12.9
3	22	9151.984	0.877	0.001	2.1
4	26	9069.625	0.827	0.035	1.1
Step2					
2	23	8992.793	0.715	---	10.7
3[a]	29	8950.85	0.719	0.035	4.3

BIC, Bayesian Information Criterion (lower values imply better model fit); LMR, Lo-Mendell-Rubin likelihood ratio test (p-value <0.05 indicates that the K_0-class model provides significantly better fit to the observed data than the preceding K_{-1}-class model); Entropy: higher values imply better classification quality.

Step 1: Selection of number of classes without including any random effects.

Step 2: Selection of the best model including random effects for intercept and slope, based on the likelihood ratio test (LRT) and the Lo-Mendell Rubin (LMR) test. Step 2 starts from the best model in Step1.

[*]Reported smallest class sizes are based on individuals' most likely class membership.

[a]The 4-class solution did not converge. Therefore, we reported the 3-class model with quadratic slope and a class-invariant random intercept and slope.

[b]Although the LMR showed a p-value >0.05, we reported the 3-class model for females in order to make it comparable to the best solution found for males.

Supplementary Table 4. Correlation matrix among all socioeconomic, psychosocial and behavioral exposures.

	Education	Occupation	Financial strain	Social connections	Social support	Social participation	Smoking	BMI	Physical activity
**Education** (elementary/high school vs. university)	1.00								
**Occupation** (manual vs. non-manual)	0.64**	1.00							
**Financial strain** (yes vs. no)	0.06	0.15**	1.00						
**Social connections** (low vs. middle/high)	0.30**	0.20**	0.1*	1.00					
**Social support** (low vs. middle/high)	0.23**	0.18**	0.15**	0.57**	1.00				
**Social participation** (low vs. middle/high)	0.17**	0.12**	-0.09	0.11**	0.21**	1.00			
**Smoking** (current/former vs. never)	-0.08**	-0.14**	0.03	-0.11**	0.00	0.08*	1.00		
**BMI** (obese/overweight vs. normal/underweight)	-0.04	-0.05	0.11*	-0.11*	-0.07*	0.01	0.08**	1.00	
**Physical activity** (inadequate vs. fitness-/health-enhancing)	0.25**	0.23**	0.04	0.33**	0.33**	0.19**	-0.06*	0.00	1.00

Significance levels for each pairwise correlation: *p<0.05; **p <0.01.

Note: most pairwise correlations were of low intensity (i.e. 0 to ±0.3), except for those between physical activity vs. social connections (0.33) and physical activity vs. social support (0.33), which were moderate, and those between education vs. occupation (0.64) and social connections vs. social support (0.57), which were strong.

Supplementary Table 5. Odds ratios (OR) and 95% confidence intervals (95% CI) for belonging to the middle/worst vs. best trajectory. Results for non-dichotomized exposures.

	Model I	Model II	Model III
	**OR (95% CI)**	**OR (95% CI)**	**OR (95% CI)**
**Socioeconomic factors**			
Education			
Elementary vs. university	2.53 (1.82-3.52)	2.40 (1.66-3.47)	2.05 (1.33-3.16)
High school vs. university	1.74 (1.36-2.22)	1.76 (1.36-2.29)	1.35 (1.00-1.82)
Occupation			
Manual vs. non-manual	1.54 (1.20-1.97)	1.20 (0.92-1.57)	1.06 (0.76-1.48)
Financial strain			
Yes vs. no	2.80 (1.95-4.02)	2.71 (1.88-3.92)	2.51 (1.59-3.96)
**Psychosocial factors**			
Social connections			
Low vs. high	2.36 (1.76-3.16)	1.93 (1.39-2.70)	1.60 (1.12-2.29)
Middle vs. high	1.53 (1.14-2.06)	1.45 (1.06-1.97)	1.27 (0.91-1.77)
Social support			
Low vs. high	1.91 (1.43-2.54)	1.29 (0.93-1.81)	1.04 (0.72-1.49)
Middle vs. high	1.28 (0.96-1.71)	1.10 (0.81-1.50)	1.08 (0.78-1.50)
Social participation			
Low vs. high	1.65 (1.12-2.43)	1.62 (1.09-2.40)	1.47 (0.96-2.24)
Middle vs. high	1.10 (0.70-1.70)	1.12 (0.71-1.75)	1.14 (0.71-1.84)
**Behavioral factors**			
Smoking			
Current vs. never	1.77 (1.32-2.35)	1.50 (1.09-2.07)	1.41 (0.98-2.02)
Former vs. never	1.12 (0.88-1.42)	1.10 (0.85-1.42)	1.12 (0.84-1.51)

BMI			
Obese vs. normal	2.62 (1.94-3.53)	2.17 (1.58-2.97)	2.20 (1.54-3.14)
Overweight vs. normal	1.12 (0.87-1.44)	1.08 (0.83-1.41)	1.15 (0.85-1.55)
Underweight vs. normal	2.50 (1.32-4.75)	2.03 (1.05-3.93)	2.00 (0.85-4.74)
Physical activity			
Inadequate vs. fitness-enhancing	7.73 (5.42-11.02)	5.91 (4.10-8.53)	4.32 (2.90-6.41)
Health-enhancing vs. fitness-enhancing	1.86 (1.31-2.63)	1.75 (1.23-2.49)	1.61 (1.10-2.34)

Model I adjusted by baseline age, and sex.
Model II adjusted by baseline age, sex and all domain-specific factors.
Model III adjusted by baseline age, sex and all other factors.
